# Causality of the Satisfaction–Performance Relationship: A Task Experiment

**DOI:** 10.5964/ejop.4075

**Published:** 2023-02-28

**Authors:** Ludmila Dudasova, Martin Vaculik, Jakub Prochazka, Petra Svitavska, Gregory Patton

**Affiliations:** 1 Department of Psychology, Faculty of Social Studies, Masaryk University, Brno, Czech Republic; 2 Department of Corporate Economy, Faculty of Economics and Administration, Masaryk University, Brno, Czech Republic; 3 Department of Economics, Accounting, and Management, Luther College, Decorah, Iowa, USA; Dublin City University, Dublin, Ireland

**Keywords:** satisfaction, performance, autonomy, need for autonomy, feedback

## Abstract

Despite the common belief among practitioners that a happy worker is a productive worker, researchers have been struggling to understand the causality between satisfaction and performance for decades. This study attempts to bring clarity to current understanding through an experiment with repeated measures of satisfaction and performance. A total of 143 participants repeatedly performed a task based on the Stroop test, with their objective performance and task satisfaction measured each time. Two different types of feedback (high/low performance) were randomly assigned to participants in order to manipulate perceived performance. The data were analyzed using a path analysis. The results support the hypothesized influence of task satisfaction on task performance and of perceived task performance on task satisfaction.

The job satisfaction–job performance relationship has attracted researchers’ attention for over 80 years, as the “Holy Grail” for industrial psychologists (Landy, 1989). Nevertheless, evidence for the happy–productive worker thesis is incomplete, with study results differing in both the strength and directionality of this relationship (see meta-analysis by Judge et al., 2001). While researchers have suggested various possible models for the relationship, their designs rarely evidence causality, as satisfaction and performance are measured concurrently, not longitudinally. Therefore, this study aims to investigate the causal relationship between satisfaction and performance, and to provide evidence for one previously proposed model of this relationship. For this purpose, an experiment was designed to objectively measure task performance and manipulate task satisfaction.

Employees’ satisfaction is essential for organizations to function. Research has shown that satisfied workers experience higher well-being (Judge & Hulin, 1993), build and maintain better relationships with colleagues (Swider et al., 2011), have greater commitment to their organization (Yoon & Thye, 2002), have lower absenteeism (Steel et al., 2002), and are less likely to leave their job (Swider et al., 2011). Ensuring conditions for high employee satisfaction is, thus, beneficial for both workers and organizations. The importance of performance is obvious: efficient workers are a source of competitive advantage for organizations (Wright et al., 1994).

Hence, the satisfaction–performance relationship is not only of academic interest but also key to creating strategic and efficient HR practices. Causality in the relationship dictates whether practitioners should seek to provide conditions for satisfaction to induce high performance, or vice versa.

## Causality of the Satisfaction–Performance Relationship

Job satisfaction represents a general attitude toward the job and the organization, with affective, cognitive, and (potentially also) behavioral components (Wright et al., 2007). General, or overall, job satisfaction is viewed as the aggregate of the satisfaction with multiple work dimensions (e.g., pay, promotion opportunities, co-workers, supervision, tasks, and the work itself) by some authors (Kinicki et al., 2002).

Performance within a job represents a broad spectrum of work-related behaviors, typically measured by their contributions toward achieving organizational goals (Campbell, 1990; Koopmans et al., 2011; Motowidlo, 2003). The individual work performance domain consists of task performance, contextual performance, and counterproductive work behavior (Koopmans et al., 2011). Traditionally, studies on performance focused mostly on task performance, which can be defined as the proficiency with which individuals perform the core substantive or technical tasks central to his or her job (Campbell, 1990).

Several meta-analyses have been conducted, yielding evidence of a significant relationship between satisfaction and performance. Judge et al. (2001) conducted a qualitative and quantitative analysis of 254 studies published during 1967–1999. The total sample included 54,471 surveyed individuals in different occupations (e.g., salesperson, scientist, teacher, manager, accountant, and nurse). The correlation between satisfaction and performance varied from -.18 to .86 across the studies, with an average corrected correlation coefficient of .30.

In the most recent meta-analysis, Davar and Bala (2012) examined 48 studies published during 1971–2008. The total sample included 2,787 workers in different professions (e.g., manager, telephone operator, teacher, and scientist). The correlation coefficient between satisfaction and performance ranged from .01 to .71, with an average corrected correlation coefficient of .30 (as in Judge et al., 2001). However, these meta-analyses described only the relationship between satisfaction and performance, but did not provide evidence about the causality of this relationship.

Regarding causality, several theoretical models for the satisfaction–performance relationship have emerged (for a review, see Judge et al., 2001) and there are also several studies that have provided limited empirical support for some of these models (see below).

### Model 1: Satisfaction Influences Performance

One of the first models to examine the satisfaction–performance relationship was based on the theory of attitudes, which assumes that an attitude towards an object is reflected in the behavior towards it (Ajzen, 1991). Performance can, thus, be perceived as a behavioral component of job satisfaction (Judge et al., 2001). Moreover, according to the broaden-and-build theory, positive emotions broaden one’s awareness and encourage novel, varied, and exploratory thoughts and actions. In turn, these broadened thought-action repertoires build intellectual, physical, social, and psychological resources for the future (Fredrickson, 2001). This theory, therefore, associates satisfaction with increased potential for high performance, as people experiencing positive emotions have better resources for performance.

Nevertheless, increased satisfaction does not always lead to higher performance. The theory of planned behavior (Ajzen & Klobas, 2013) suggests that behavior is influenced by not only the attitude itself but also one’s ability to behave accordingly and the views of significant others about the object (Ajzen & Klobas, 2013). As the influence of the aptitude and significant others´ opinions may be stronger than the attitude itself, change in the attitude (satisfaction) does not necessarily cause change in the behaviour (performance).

The positive influence of satisfaction on performance has been supported by only a few studies. Riketta (2008)’s meta-analysis of 11 research papers from 1974–2006 generated a sample of 3,077 workers. All 11 studies used a panel (i.e., longitudinal) design with repeated measurements of job attitudes and performance, with first and second measurements separated by between one month and 18 months. The correlation between satisfaction and performance varied from -.03 to .66 across the studies, and the general effect of satisfaction on subsequent performance was very weak but statistically significant (β = .03, *p* < .05). This effect is significantly weaker than the usually reported correlation between satisfaction and performance, therefore the unexplained part of the correlation can be potentially explained by the opposite causal relationship. Moreover, as the author himself stated, panel design does not have potential to provide irrefutable evidence for causality.

### Model 2: Performance Influences Satisfaction

According to expectancy theory (Isaac et al., 2001), performance leads to internal and external rewards (salary, joy of doing well, etc.) that are satisfactory (Lawler & Porter, 1967). In addition, self-determination theory (Deci & Ryan, 2008) posits that satisfaction follows from the rewards resulting from behavior, though behavioral motivations also influence this process. However, satisfaction is affected by not only procured rewards but also important job characteristics (Hackman & Oldham, 1976), significant others’ opinions about the job (O’Reilly & Caldwell, 1985), and the worker’s affectivity (Dormann & Zapf, 2001). Consequently, how performance influences satisfaction may be difficult to detect due to other intervening variables.

The influence of performance on satisfaction was mainly investigated in early studies and lacks convincing empirical support. For example, Brown and Peterson (1994) found a significant correlation between the two constructs, but the effect was reduced by the relative influence of other variables or mediated through other constructs; an *r* = .31 (*p* < .01) correlation became an *r* = .04 (*p* > .05) performance → satisfaction path coefficient when effort and role conflict were also modeled to influence job satisfaction. Christen et al. (2006) analyzed data collected by Lusch and Serpkenci (1990) from 177 US grocery store retailers and highlighted the importance of the measures used. With an aggregate measure of performance (including effort and ability), they identified an insignificant effect of job performance on satisfaction ($\hat{\beta }$ = .02, *p* > .1), whereas with a narrow measure of performance (without effort), they identified a slightly higher positive effect ($\hat{\beta }$ = .14, *p* = .06), though still much smaller than the effect reported by Lusch and Serpkenci ($\hat{\beta }$ = .29, *p* < .01). In Riketta’s (2008) meta-analysis, the effect of job performance on satisfaction was negative (β = -.08, *p* < .001) and only significant in studies with a moderate time lag between measurement waves (7–12 months). The ambiguity of these prior findings may result from the different influences of various external variables in longitudinal surveys (Taris & Kompier, 2003).

### Model 3: Satisfaction and Performance Are Reciprocally Related

The model for the reciprocal relationship between satisfaction and performance has no distinct theoretical foundation, but rather synthesizes the two preceding models (Judge et al., 2001). Evidence for a mutual causal relationship was offered by Downey et al. (1975)’s longitudinal study of 94 managers and manual workers. In another longitudinal study of 80 telephone-company employees, Wanous (1974) found that satisfaction and performance influenced each other. Evidence for validity of this model was provided also by recent research (e.g. Alessandri et al., 2017; Bakotić, 2016; Yang & Hwang, 2014). The results from Riketta (2008)’s meta-analysis suggest that satisfaction is more likely to influence performance than vice versa: the weak but significant effect of satisfaction on performance (β = 0.03) was accompanied by almost no statistically significant evidence for the reverse causal direction.

## Limitations of the Current Evidence on Causality

The main limitation of current evidence on causality in the satisfaction–performance relationship is most studies’ research design. Satisfaction and performance are often measured concurrently and rarely longitudinally, thus limiting the possibility of determining causality (Judge et al., 2001). Although several longitudinal studies were included in Riketta (2008)’s meta-analysis, their non-experimental design cannot provide clear evidence of causality in this relationship. Riketta (2008) inferred the causal influence of job attitudes on performance from the significance of correlations between job attitudes at the first measurement and performance at the second. However, we consider this approach suboptimal for inferring causal relationships without raising serious doubts about the interpretation of results. There are at least two alternative explanations for the correlation between job attitudes and performance measured in two separate measurement waves. The first explanation is that a relatively stable third variable might influence both constructs in both measurements. For example, personality trait conscientiousness predicts both job satisfaction (Judge et al., 2002) and performance (e.g., Prochazka et al., 2018) The second explanation is linked to the selection criteria for studies included in Riketta (2008)’s meta-analysis: “No major changes in the work environment, such as an organizational merger or a change in the task of the participants, occurred between the measurement waves” (p. 473). With no major changes in the work environment, there was also no reason for satisfaction to vary except to the extent caused by change in performance. If context remained the same, then satisfaction also remained similar, and so the influence of change in satisfaction on performance could not be found.

We are, consequently, convinced that experiments are needed to provide compelling evidence of causal relationships.

## Satisfaction and Performance in Experiment

To test the reciprocal causal relationship between job satisfaction and job performance, both constructs need to be manipulated. However, the complexities of these constructs make this virtually impossible in an experimental study. Therefore, we focused on the key dimensions of satisfaction and performance—task satisfaction and task performance.

### Task Satisfaction

Job satisfaction is typically measured with self-report questionnaires addressing overall satisfaction or satisfaction with individual job aspects (e.g., task satisfaction, satisfaction with supervision). The correlations between global and facet job satisfaction scales suggest that task satisfaction may be the most important contributor to general job satisfaction (Ironson et al., 1989; Skalli et al., 2008; Taber & Alliger, 1995). Additionally, satisfaction with work content has previously been found better correlated with task performance than other job satisfaction components (Edwards et al., 2008). Therefore, our experiment focuses on influencing and measuring task satisfaction as the most important dimension of job satisfaction. We are aware that a job comprises of several different tasks. Our focus on satisfaction with a single task is a simplification typical of laboratory experiments.

### Objective Task Performance

The studies focused on job performance usually differentiate between task performance, contextual performance and counterproductive work behavior and measure one or more of these dimensions (e.g., Koopmans et al., 2011). All dimensions of job performance are mostly evaluated by subjective subordinate, supervisor, peer, or self-ratings using a questionnaire (Conway & Huffcutt, 1997; Heidemeier & Moser, 2009).

There are various sources of error in subjective performance ratings: cognitive heuristics, social desirability, personal incentives, the evaluator’s personality, etc. (for more details, see Adler et al., 2016; Bol & Smith, 2011; Grund & Przemeck, 2012; Landy & Farr, 1980; Prendergast & Topel, 1993). Consequently, many evaluators are needed to ensure sufficiently reliable subjective performance measures. This is difficult to achieve in research studies, and although combining different evaluators increases the value of the intrinsic consistency coefficient, it also decreases the construct validity of performance measurement (Mount et al., 1998).

Some researchers attempt to avoid subjective biases by employing objective criteria (e.g., work results) to measure performance (e.g., Crant, 1995). Although not itself a result (Koopmans et al., 2011), the performance influences how the work is done and if the goals are achieved. Therefore, work result or the level of goal accomplishment can be used as an estimate of job performance, specifically task performance.

Despite the use of objective indicators, the results of the work may result in a biased evaluation of work performance in some situation, similar to the subjective evaluation by a questionnaire. The objectively measured results reflect the workers’ behavior but might be also influenced by various external factors (Steel & Mento, 1986). In field research, it is difficult to identify a sufficiently large sample of people performing the same tasks under the same conditions (e.g., with same support from co-workers, same leadership style of supervisors, in same region, with same internal or external clients). Consequently, the conditions and the context might strongly influence the measured job outcomes.

Highly reliable performance measurement requires the same working conditions for all participants. Such conditions can be created in laboratory experiments. However, measuring performance in an experiment introduces its own constraints (Dipboye & Flanagan, 1979), such as limited ecological validity, the impossibility of capturing contextual performance and counterproductive behavior, and the inability to investigate whether performance can be maintained long term. Nevertheless, these limits connected to the ecological validity are counterbalanced by high internal validity of such experiments. That is why experiments are sometimes used to explore and understand the influence of various variables on performance (e.g., Cadsby et al., 2007; Fatseas & Hirst, 1992; Hu & Kaplan, 2015; Landers et al., 2017; Vancouver et al., 2001) and to complement field studies which have higher ecological but lower internal validity.

The satisfaction–performance relationship has been extensively studied in real conditions (e.g., MacKenzie et al., 1998; Wright & Cropanzano, 2000), but experiments and the use of objective performance indicators are rare. We, therefore, decided to investigate the satisfaction–performance relationship in laboratory conditions with objective performance indicators, making a critical and controllable first step toward understanding these constructs’ causal relationship.

Our study focuses on task performance, because of three reasons. It is the most often investigated component of performance, especially in experimental studies (e. g., Johnson et al., 1993; Simon, 2006), previous studies showed that it explains the largest portion of variance in overall performance (Conway, 1999; Motowidlo & Van Scotter, 1994; Rotundo & Sackett, 2002), and it is possible to measure it using objective criteria.

In this study, we use an experimental design with objective performance measurement to test both possible directions of the satisfaction–performance relationship, hypothesized as follows:

Hypothesis 1: Task satisfaction positively affects objective task performance.

Hypothesis 2: Perceived task performance positively affects task satisfaction.

## Method

### Procedure

We conducted our experiment in a university computer lab. Table 1 presents the experiment design.

**Table 1:** Design of the Experiment

Step	Description
*1*	informed consent, need for autonomy assessment, instructions
*2*	autonomy manipulation (A+/A-)
*3*	training
*4*	task satisfaction measurement (related to training) 0
*5*	task performance measurement 1
*6*	perceived performance manipulation (i.e. feedback; F+/F-) 1
*7*	task satisfaction measurement 1
*8*	task performance measurement 2
*9*	perceived performance manipulation (F+/F-) 2
*10*	task satisfaction measurement 2
*11*	task performance measurement 3
*12*	perceived performance manipulation (F+/F-) 3
*13*	task satisfaction measurement 3
*14*	demographic variables
*15*	autonomy manipulation check
*16*	debriefing

*Note.* A+ = autonomous conditions, A- = non-autonomous conditions, F+ = positive feedback, F- = negative feedback.

Participants were invited to complete three iterations of a task inspired by the Stroop test (Stroop, 1935). With a total duration of approximately 20 minutes, the experiment involved initial training followed by three tasks each comprising three parts. In each part, participants were asked to determine as quickly as possible the color (red or blue, red or green, and blue or green) of 30 words randomly displayed on a computer screen. Their speed and error rate were both measured.

Participants repeated this task three times because we wanted to determine whether the satisfaction–performance relationship would vary across measurement waves. We also assumed that positive perception of a new task, considered interesting and satisfying regardless of the experimental conditions, could bias the results; repetition served to avoid this. We considered the experiment’s duration to be sufficient since attitudes can change relatively quickly (Rydell & McConnell, 2006).

To achieve sufficient variability in participants’ initial task satisfaction, we manipulated it via autonomy, as a significant antecedent of satisfaction (see, e.g., Hackman & Oldham, 1975). Such manipulation also allowed us to observe the causal influence of satisfaction as a mediator between a satisfaction antecedent and performance. Job autonomy is “the degree to which a job provides substantial freedom, independence, and discretion to the employee in scheduling the work and in determining the procedures to be used in carrying it out” (Hackman & Oldham, 1975, p. 162). Previous research supports the impact of job autonomy on satisfaction (e.g., Fahr, 2011; Schjoedt, 2009; Skaalvik & Skaalvik, 2014).

Accordingly, participants were randomly assigned to groups with and without autonomous working conditions. Because some participants may prefer non-autonomous conditions, we asked all participants if they preferred autonomous or non-autonomous conditions (reflecting their actual need for autonomy) immediately after describing the experiment to them. We tested the effect of need for autonomy in later statistical analyses.

After manipulating autonomy and training, initial task satisfaction was assessed using a questionnaire. Participants then completed the first task, in which their performance was measured. On completion, they received randomly assigned positive or negative feedback on their task performance (manipulation of perceived performance). We used written computer-mediated feedback to ensure feedback consistency across treatments.

Participants’ task satisfaction was then assessed. This process of measured task completion and subsequent random performance feedback was repeated twice by each participant. Participants were then asked to provide their demographic information and their perceived task autonomy was assessed (i.e., the task autonomy manipulation check). After all the data were collected, a debriefing was held at which participants were told the study’s true purpose.

The laboratory environment ensured a constant value for potentially intervening variables. The task was administered in the presence of a trained administrator, who could provide answers to participants’ questions or offer technical support if needed. The study was conducted in accordance with the Ethics Code of the American Psychological Association (2017) including informed consent, respect for anonymity and final debriefing.

#### Manipulation of Autonomy

Half the participants were randomly assigned to the autonomous condition: they could choose the order of the tasks (which two colors they wanted to distinguish first and then second) and decide when the task started. The other half of participants were assigned to the non-autonomous condition, whose color combinations in the tasks were randomly sequenced by the computer and who had to wait for each task to run automatically. Both groups had the same set of tasks, which tested the same competencies.

#### Manipulation of Perceived Performance

Since participants could not estimate how well they performed in the Stroop test, it was possible to manipulate perceived performance through random feedback. Half the participants were informed after each of the first and second tasks that they had performed above the group average; the other half were informed at the same time points that their performance on each task was below the group average. As we wanted to observe also an effect of perceived performance change, all participants received opposite performance feedback to that given following the first and second tasks on completing the third task (i.e., below average for the first group; above average for the second group).

### Participants

The sample comprised 143 Czech adults—52 (36.36%) men and 91 (63.64%) women—recruited via an invitation on the university’s information system, its official Facebook page, and in leaflets distributed on campus. Consequently, most participants were students. Participants studied or worked in the following fields: social sciences (62, 44.4%), law (8, 5.6%), education (7, 4.9%), economics (17, 11.9%), philology (10, 7.0%), technical sciences (4, 2.8%), informatics (8, 5.6%), medicine (5, 3.5%), and another area or multiple areas (20, 14.0%). Two participants (1.4%) did not report their field of study or work. Participants ranged in age from 18 to 30 years (*M* = 22.83, *SD* = 2.11).

To avoid revealing the study’s true purpose, participants were initially told that its goal was to compare the ability of men and women in distinguishing colors. We considered students’ results to be fairly generalizable to the general population, but excluded psychology students and graduates (who were distrustful of the stated research purpose in prior pilot study) to ensure the experiment’s true purpose remained unknown (until the debrief). No participant reported having any form of diagnosed color blindness, so all were able to distinguish colors in the tasks.

### Measures

#### Performance

Performance was measured as the time taken to complete the Stroop test task. We calculated the total time for completing the three parts to determine performance in each of the three measurement waves. The error rate was also measured to statistically control for the effect of fast but low-quality performance. Participants could not judge how well they were performing because they received no objective feedback on the time and quality of their own or peers’ performance.

#### Satisfaction

The widely recognized short version of the Job Diagnostic Survey’s General Satisfaction Scale (Hackman & Oldham, 1974) was used to assess task satisfaction. It comprises three self-evaluating statements, rated on a 5-point Likert scale (1 = Do not agree, 5 = Agree). The items were adjusted to measure task satisfaction: e.g., the original statement “*I frequently think of quitting this job”* was changed to *“I often considered abandoning the task.”* The inventory’s clarity was verified in a pilot study comprising ten participants with different socio-demographic characteristics. Prior analysis of the adjusted Czech version of this measure (Vaculik et al., 2016) found high internal consistency (α = 0.95) and strong factor loadings (0.81; -0.74; 0.84). Satisfaction was measured a total of four times (i.e., after the initial training and after each of the three tasks).

#### Need for Autonomy

We measured participants’ need for autonomy through a single question on whether they preferred to work in autonomous conditions Only 33 (23.08%) participants preferred autonomous task conditions; the majority (110, 76.92%) preferred non-autonomous conditions. Participants were randomly assigned to experimental groups regardless of their autonomy preference, with those not assigned to their preferred conditions informed that their preference was oversubscribed. We monitored the interaction of autonomy and need for autonomy, expecting autonomy to only increase satisfaction in participants with high need for autonomy. The uneven distribution of participants with high or low need for autonomy was unproblematic in the experiment as autonomy was mainly included to achieve variability in task satisfaction. Variation in need for autonomy and the autonomy of tasks also helped to simulate a real working environment where people work under different conditions.

#### Perceived Autonomy (Manipulation Check)

Perceived task autonomy was assessed at the end of the experiment by three items from Hackman and Oldham’s (1974) Job Diagnostic Survey, adjusted to the following: *“The task did not provide an opportunity to use my own initiative and judgment”*; *“The task provided an opportunity to work freely without important restrictions”*; and *“The task allowed me to decide how and when to do it.”* Participants rated each item on a 5-point Likert scale (1 = Do not agree, 5 = Agree).

## Results

We tested the hypotheses using single composite indicators for satisfaction and performance in each measurement wave. The hypotheses could not be tested within a full structural model due to the high number of parameters and relatively small sample size determined by the experimental design. The model contained four dichotomous predictors: perceived performance, as manipulated by feedback following each of the three tasks (0 = negative/negative/positive; 1 = positive/positive/negative); need for autonomy (0 = no; 1 = yes); autonomy (0 = no; 1 = yes); and whether autonomy corresponded with need for autonomy (0 = no; 1 = yes).

Verifying the success of our attempt to manipulate task autonomy, participants in autonomous conditions reported perceiving greater task autonomy at the end of the experiment compared to participants in non-autonomous conditions (*M_na_* = 9.56, *SD_n_*_a_ = 3.35, *M_a_* = 11.03, *SD_a_* = 2.79, *t* = -2.68, *p* < .01, Cohen’s *d* = .48).

Performance was operationalized as the completion time for each of the three tasks. The error rate (i.e., number of mistakes in a task) was only checked to control for participants who worked quickly but inaccurately. In the first task, participants who worked slowly also made more errors (Spearman's rho = .46, *p* < .01). Further, performance measured through completion time did not correlate with the number of errors in the second (Spearman's rho = .03, *p* = 70) and third tasks (Spearman's rho = -.03, *p* = 77). Since the number of errors did not distort performance operationalized as task completion time, it was not included in subsequent analyses, thereby also avoiding the problems of a variable with low variability and very skewed distribution.

Table 2 presents the descriptive statistics and Pearson correlations for all the variables. Autonomy conditions were negatively related to the total completion time for each task (i.e., participants were faster in autonomy conditions), although this relationship weakened in each measurement wave. Neither need for autonomy nor the interaction of autonomy and need for autonomy were found to be related to any of the four measurements of satisfaction. Strong interrelationships were found between the four measurements of satisfaction and between performance in all three tasks. The relationship between satisfaction and the time needed to complete the following task (i.e., performance) was negative (i.e., more satisfied participants worked faster), but weakened in each measurement wave. The Cronbach’s alpha values showed good internal consistency of performance measures for the three sub-tasks administrated in each measurement wave (see values on the diagonal of Table 2).

**Table 2:** Descriptive Statistics and Correlations

Variable	M	SD	1	2	3	4	5	6	7	8	9	10
1. Autonomy	0.50	0.50										
2. Need for autonomy	0.26	0.44	-.03									
3. Feedback	0.52	0.50	-.01	.01								
4. Satisfaction0	23.74	4.23	.00	.08	.01	(.73)						
5. Satisfaction1	25.21	4.21	.12	-.02	.32**	.55**	(.71)					
6. Satisfaction2	24.90	4.05	-.07	-.03	.19*	.57**	.70**	(.64)				
7. Satisfaction3	24.20	4.28	-.11	.03	-.09	.64**	.46**	.67**	(.63)			
8. Performance1	73.99	21.89	-.35**	.01	.03	-.32**	-.19*	-.07	-0.16	(.84)		
9. Performance2	58.50	12.64	-.30**	-.07	.11	-.33**	-.18*	-.11	-.27**	.86**	(.90)	
10. Performance3	56.69	12.28	-.22*	-.04	.13	-.27**	-.12	-.06	-.24**	.75**	.92**	(.85)

*Note.* Cronbach alphas are in parentheses; the measure of performance was time, lower value means faster performance.

*p < .05. **p < .01.

The internal consistency of the task satisfaction measure was above .70 for the first and second measurement waves. However, in the third and fourth measurement waves, Cronbach’s alpha values were between .60 and .70., presumably due to the reverse-coded item 2 (i.e., *“I often considered abandoning the task*). We assume that responses to this item reflected satisfaction with not only the task itself but also the length of the experiment and the repeated administration of the same task and questions. Though Cronbach's alpha values nonetheless remained satisfactory for all the analyses, lower internal consistency in the third and fourth measurement waves should be considered when interpreting the results.

### The Path Model

The hypotheses were tested within a complex model using the path analysis method in MPLUS 6.0 (Muthén & Muthén, 2011), employing maximum likelihood estimation with robust standard errors (MLR). The path analysis allowed simultaneous testing of all the hypothesized relationships. This approach was chosen to reduce the number of analyses, and thus decrease the risk of false positive results. We also wanted to control for the possible influence of variables that might affect the tested relationships.

Our model was inspired by the three-wave model proposed by Cole and Maxwell (2003) for studies using longitudinal data from large surveys, with all the variables measured concurrently in repeated measurement waves. However, our study was an experiment in which different variables were measured successively and not all within one questionnaire. Our model reflects that satisfaction was measured both before and after performance. Our sample was smaller than that typical of longitudinal surveys, which prevented use of a full structural model.

**Figure 1:**
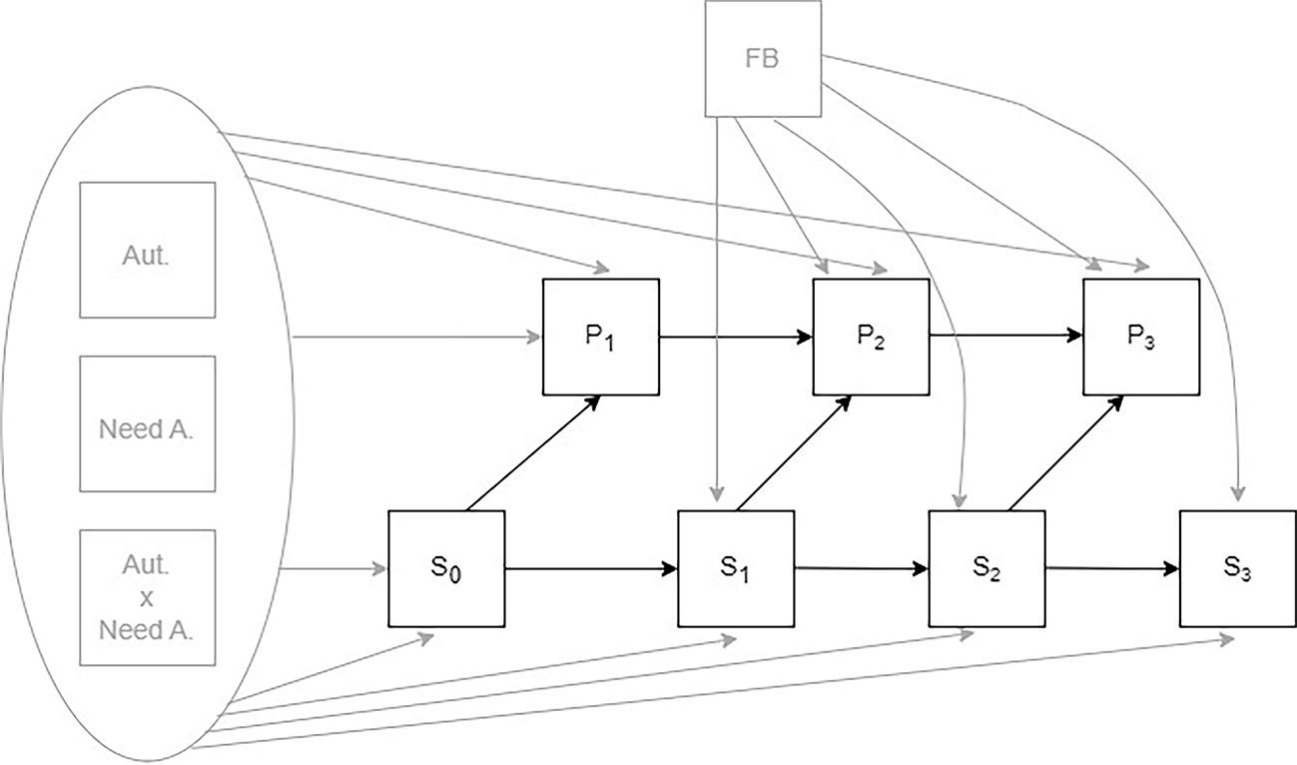
Tested Model of Influence of Satisfaction on Performance and Perceived Performance (FB) on Satisfaction *Note.* Autonomy (Aut.), Need for Autonomy (Need A.) and their interaction are modeled as three independent variables. A direct path from each of them leads to each measurement wave of satisfaction (S_0_, S_1_, S_2_, S_3_) and performance (P_1_, P_2_, P_3_). One grey arrow from a pack of variables represents three paths (one from each of the variables) in order to enhance the figure’s clarity.

We modeled post-task satisfaction as predicted by pre-task satisfaction (S_0_ → S_1_ → S_2_ → S_3_). Similarly, we modeled performance (i.e., completion time) in each task as predicted by performance in the previous task (P_1_ → P_2_ → P_3_). Performance in each task was regressed on pre-task satisfaction (S_0_ → P_1_; S_1_ → P_2_; S_2_ → P_3_). The path between performance and subsequent satisfaction remained closed because participants did not receive information about their real performance. Information about performance in each task was manipulated through randomly assigned feedback. Consequently, we regressed satisfaction on the type of feedback participants received before their satisfaction was measured. Since there were only two variations in the type of feedback for participants after all three sets of tasks, all three measurements of satisfaction (S_1_, S_2_, S_3_) were regressed on the same dichotomous predictor. Autonomy, need for autonomy, and their interaction were included in the model to control for their effect. All four satisfaction measurements and all three performance variables were regressed on autonomy, need for autonomy, and their interaction. Figure 1 depicts the full model. Besides the direct paths between variables, indirect paths to assess mediation effects were also tested.

We first assessed how the model fitted the data (χ^2^(15) = 55.06, *p* < .01; RMSEA = .15; 90% CI_RMSEA_ = .11, .19; CFI = .95). The CFI showed a very good fit according to the cut-off criterion of Hu and Bentler (1999). Conversely, RMSEA—one of the most commonly used model fit indicators—suggested a poor fit. However, RMSEA is not a reliable indicator for models with a small number of degrees of freedom because it often falsely indicates a poor fit (Kenny et al., 2015). According to modification indexes, the model would be improved by opening direct paths between the first and last satisfaction measurements. However, this step lacked a theoretical basis and the model fit was sufficient to test the hypotheses.

For performance, the model explained 23% of the variability in the first task (p < .01), 76% in the second task (p < .01), and 85% in the third task (p < .01). For satisfaction, it explained only .01% of the variability before the first task (*p* = .64), 41% after the first task (*p* < .01), 52% after the second task (*p* < .01), and 51% after the third task (*p* < .01). Table 3 provides a decomposition of effects from the path analysis. For each variable, direct effects are listed in the first row, with the sum of the indirect effects resulting from mediation analyses in the second row.

**Table 3:** Decomposition of Effects From Path Analysis

	→Satisfaction0				→Satisfaction1				→Satisfaction2				→Satisfaction3				→Performance1				→Performance2				→Performance3			
Variable	Est.	*SE*	*p*	St. est.	Est.	*SE*	*p*	St. est.	Est.	*SE*	*p*	St. est.	Est.	*SE*	*p*	St. est.	Est.	*SE*	*p*	St. est.	Est.	*SE*	*p*	St. est.	Est.	*SE*	*p*	St. est.
Autonomy	.01	.90	.99	.00	1.19	.74	.11	.14	-1.45	.60	.02	-.18	-.52	.67	.44	-.06	-18.2	4.01	<.01	-.42	.77	1.27	.54	.03	1.04	.89	.24	.04
indirect					.01	.49	.99	.00	.84	.63	.18	.11	-.45	.63	.47	-.05	-.02	1.49	.99	-.00	-9.19	2.57	<.01	-.37	-7.56	2.77	<.01	-.31
Need for autonomy	.69	1.12	.54	.07	-.14	.70	.85	-.01	-.60	.72	.41	-.07	.56	.72	.44	.06	-4.71	4.80	.33	-.09	-.88	1.59	.58	-.03	-.23	1.14	.84	-.01
indirect					.38	.62	.54	.04	.17	.72	.81	.02	-.32	.82	.70	-.03	-1.14	1.90	.55	-.02	-2.37	2.45	.33	-.08	-2.97	2.51	.24	-.11
Autonomy*Need	.09	1.60	.96	.01	-.88	1.00	.38	-.07	.90	.96	.35	.07	-.11	1.07	.92	-.01	12.35	7.83	.12	.18	-2.76	2.24	.22	-.07	1.73	1.76	.33	.34
indirect					.05	.87	.96	.00	-.59	.97	.54	-.05	.23	1.14	.84	.02	-.15	2.63	.96	-.00	6.26	4.00	.12	.16	3.10	3.70	.40	.08
Feedback					2.63	.58	<.01	.31	-.35	.61	.57	.04	-1.95	.54	<.01	-.23					2.60	1.11	.02	.10	.58	.84	.49	.02
indirect									1.86	.53	<.01	.23	1.14	.45	.01	.13					-.41	.35	.24	-.02	2.55	1.14	.03	.10
Satisfaction0					.54	.08	<.01	.55									-1.65	.46	<.01	-.32								
indirect									.40	.06	<.01	.40	.29	.05	<.01	.29					-.06	.06	.17	-.03	.05	.04	.25	.02
Satisfaction1									.71	.10	<.01	.74									-.16	.12	.20	-.05				
indirect													.53	.09	<.01	.52									.08	.07	.24	.03
Satisfaction2													.42	.06	<.01	.71									.12	.10	.22	.04
indirect																												
Performance1																					.50	.06	<.01	.86				
indirect																									.45	.10	<.01	.80
Performance2																									.91	.11	<.01	.94

*Note.* Rows with the variable name contain only direct effects; find sum of indirect effects through various mediators in the rows “-indirect”. The measure of performance was time, negative coefficient means positive influence on performance.

Autonomy did not influence satisfaction, but it had a direct negative effect on the completion time of the first task (i.e., a positive influence on performance) and an indirect effect on performance in the second task (see the second row in the last but one column in Table 3 for the total indirect effect). This indirect effect consisted of three parts, the only significant mediation was through performance in the first task (partial indirect effect: A → P_1_ → P_2_: Est. = -9.00, SE = 2.56, *p* < .01, St. est. = -.36). The mediations through satisfaction were negligible.

Autonomy had also an indirect effect on performance in the third task (see the second row, the seventh column in Table 3 for the total indirect effect). This indirect effect consisted of five parts, but the only relevant was the one through performance in the first and second task (partial indirect effect: A → P_1_ → P_2_ → P_3_: Est. = -8.19, SE = 2.97, *p* < .01, St. est. = -.34). Neither need for autonomy nor its interaction with autonomy significantly affected satisfaction or performance in any measurement wave (either directly or indirectly, see the third to sixth rows in Table 3).

### Hypotheses Testing

#### The Influence of Satisfaction on Performance

As Table 1 reports, initial satisfaction (S_0_) correlated negatively with all three performance measurements. Because performance was measured as a time, a negative correlation means that initial satisfaction positively influenced later performance measurements. However, the influence of satisfaction on performance became weaker for the second and third measurements of satisfaction: satisfaction after the first task (S_1_) correlated only weakly with performance in the second task (P_2_), and satisfaction after the second task (S_2_) did not correlate significantly with performance in the third task (P_3_).

The significant influence of initial satisfaction (S_0_) on performance in all three tasks can also be seen in the path model. Participants who were more satisfied before the first task completed it faster than their less-satisfied peers (see the negative path in the nineth row, the fifth column in Table 3). Initial satisfaction (S_0_) was also indirectly linked to better performance in subsequent tasks (i.e., P_2_ and P_3_). The influence of S_0_ on P_2_ and P_3_ was mediated mainly by performance in the first task (P_1_). Therefore, more satisfied participants who completed the first task faster than less satisfied participants were also quicker to complete the second task (partial indirect effect: S_0_ → P_1_ → P_2_: Est. = -.82, SE = .30, *p* < .01, St. est. = -.27) and third task (partial indirect effect: S_0_ → P_1_ → P_2_ → P_3_: Est. = -.74, SE = .34, *p* = .03, St. est. = -.26). Initial satisfaction had a weak-to-moderate effect on performance in all subsequent tasks, thus supporting Hypothesis 1’s prediction that task satisfaction positively influences objective task performance.

However, fluctuation in satisfaction between measurement waves did not significantly influence performance in the following tasks (i.e., the direct effects of S_1_ and S_2_ on P_2_ and P_3_ were small and insignificant; see Table 3).

Contrary to our expectation, autonomy did not influence satisfaction, so there was no observable influence of autonomy on performance through satisfaction. With reference to Baron and Kenny (1986), the condition for mediation analysis that the predictor (i.e., autonomy) should influence the mediator (i.e., satisfaction) was not met.

We could, however, test the indirect effect of another predictor of satisfaction on performance. Perceived performance (manipulated by feedback, FB) positively influenced task satisfaction after the first task (S_1_), which, in turn, influenced performance in the second task (P_2_). However, the indirect effect of perceived performance on later task performance through satisfaction was not significant. The increase in satisfaction caused by perceived performance did not cause a further increase in objective performance (the eighth row, the sixth column in Table 3). Finally, perceived performance (i.e., manipulated by feedback) in the first task influenced objective performance in the second task only directly (the seventh row, the sixth column in Table 3).

#### The Influence of Perceived Performance on Satisfaction

As Table 4 reports, initial satisfaction (S_0_) was similar in both performance feedback groups. After the first manipulation of perceived performance, the group with positive feedback became more satisfied with the task than the group with negative feedback (S_1_). Participants in the group with positive feedback also remained more satisfied after receiving positive feedback after the second task (S_2_). With each group receiving opposite feedback after the third task compared to their feedback following the first and second tasks, final satisfaction after the third manipulation of perceived performance was similar in both groups (S_3_).

**Table 4:** Differences in Satisfaction in Groups Divided According to the Perceived Performance Manipulation

Group	Feedback	*M*	*SD*	*t*(123)	*p*
**Satisfaction0**					
	Negative/Negative/Positive	23.68	4.25	-.13	.89
	Positive/Positive/Negative	23.78	4.25		
**Satisfaction1**					
	Negative/Negative/Positive	23.83	4.33	-3.68	< .001
	Positive/Positive/Negative	26.48	3.70		
**Satisfaction2**					
	Negative/Negative/Positive	24.08	4.47	-2.19	.03
	Positive/Positive/Negative	25.65	3.50		
**Satisfaction3**					
	Negative/Negative/Positive	24.60	4.37	1.01	.32
	Positive/Positive/Negative	23.83	4.19		

The influence of perceived performance can also be seen in the path model. High perceived performance (manipulated by random performance feedback) was a weak-to-moderate positive predictor of satisfaction. Positive feedback led to higher satisfaction after both the first and second tasks; since the same participants received positive feedback after both tasks, the effect of P_1_ on S_2_ was mediated by S_1_ (the eighth row, the third column in Table 3). This positive influence of the first two sets of positive feedback remained even after receiving negative feedback following the third task (the eighth row, the last column in Table 3). However, the negative direct effect of the negative feedback and the positive indirect effects of the previous two sets of positive feedback were similarly large, leaving the total effect of perceived performance manipulation on satisfaction at the end of the experiment (S_3_) close to zero. Compared to the negative/negative/positive feedback group, the group with positive/positive/negative feedback had higher task satisfaction after the first two tasks, but similar task satisfaction after the third task. Perceived performance manipulated by feedback influenced satisfaction after each task. These results support Hypothesis 2’s prediction that high perceived performance in a task positively influences task satisfaction.

## Discussion

Our laboratory experiment provides new evidence of reciprocal causality in the task satisfaction–task performance relationship. Specifically, the influence of perceived performance on subsequent satisfaction was more strongly supported than the influence of satisfaction on objective performance. Participants in the positive/positive/negative feedback group had higher task satisfaction after the first and second tasks compared to the negative/negative/positive feedback group. This difference was found despite participants being asked to rate satisfaction with the task, rather than with their performance. After each group received opposite feedback following the third task (compared to their feedback following the first and second tasks), there were no significant differences between the groups in subsequent assessment of task satisfaction. According to the mediation analysis, the positive effect of the first two sets of feedback persisted and increased the first group’s task satisfaction. Additionally, a new effect of the third (opposite) set of feedback appeared, which reduced satisfaction in the first group and increased it in the second group. Therefore, the results indicate that a person who thinks they are performing well is inclined to be more satisfied with the task than a person who thinks they are not performing well.

We also found support for the influence of satisfaction on performance. Participants with higher task satisfaction at the beginning of the experiment performed better in all three tasks compared to participants who expressed lower initial task satisfaction. Nevertheless, only initial satisfaction influenced subsequent performance, and this impact gradually declined with each further task. Later changes in satisfaction did not lead to a change in performance, either directly or indirectly. This could be due to the small variability in satisfaction between individual measurement waves, which gave limited room for finding a statistically significant relationship. The importance of personal or dispositional determinants of job attitudes, and therefore their consistency over time and across situations is supported by longitudinal evidence and discussed by Staw and Ross (1985). Nevertheless, some variability in satisfaction was achieved by manipulating perceived performance. Although this manipulation affected satisfaction, it did not thereby influence objective performance in subsequent tasks. Therefore, participants persuaded they had performed well were more satisfied, but this did not raise their subsequent performance. One plausible explanation is that participants who thought they were performing well perceived no need to change their work approach and increase their efforts, perhaps believing that their current approach would also be sufficient for good performance in the future. This result suggests that the reciprocal influence of satisfaction on performance may not be an endless cycle. We assume that another part of satisfaction unrelated to performance (e.g., influenced by task characteristics or mood) affected work approach and, therefore, also task performance.

Our experiment also manipulated task autonomy to ensure greater variability in satisfaction and to observe the possible impact of satisfaction in mediating the autonomy–performance relationship. However, the conditions for mediation analysis were not met (Baron & Kenny, 1986), as the independent variable (autonomy) did not relate to the mediator (satisfaction). Participants with autonomous task conditions did not have higher task satisfaction compared to peers without autonomous task conditions, despite successfully manipulating autonomy (according to the manipulation check) and despite controlling for participants’ need for autonomy. We can explain this result by referring to Dodd and Ganster (1996), who reported that autonomy increases job satisfaction in high skill-variety conditions, but only has a negligible influence thereon in low skill-variety conditions. We can, therefore, expect the link between autonomy and satisfaction to be stronger if participants are assigned another task requiring them to use different skills. However, autonomous conditions had a relatively strong influence on performance in all three tasks, even though the option to choose when to start and in which order to perform the (identical) tasks did not make them easier to complete, and so could not directly influence the outcome. Therefore, autonomous conditions indirectly led participants to achieve higher performance. The mediator could have been another attitude or feeling not measured in this experiment (e.g., engagement or involvement).

Our inability to monitor the possible impact of autonomy on performance through satisfaction somewhat weakens our evidence of the influence of satisfaction on performance. The relationship between initial satisfaction and later performance might be influenced by participants’ beliefs about their abilities to succeed in the task. Participants could anticipate their likely performance in the task according to the task description and training, in turn affecting their satisfaction. For example, participants who knew they could concentrate well and were generally successful in speed tasks might have had higher task satisfaction than participants who were less confident in speed tasks. If so, then the higher performance of more satisfied participants would be also an outcome of their anticipated performance. However, anticipated performance is only another predictor of satisfaction besides autonomy and this interpretation does not question the very effect of satisfaction on performance. Moreover, we measured task satisfaction before participants undertook the task in our experiment. As the chosen task was very atypical, it is unlikely that participants were able to accurately predict their future performance. Therefore, this study provides stronger evidence of the influence of satisfaction on performance than do prior longitudinal studies that focused on overall job satisfaction and job performance, where satisfaction in the first measurement might be influenced by previous job performance. Nevertheless, we recommend conducting another experiment to manipulate a different antecedent of satisfaction and examine the causal effect of this predictor on task performance through satisfaction. Such an experiment would provide even clearer evidence of the impact of satisfaction on performance.

Interestingly, the described effects of performance on satisfaction are inconsistent with the findings of Riketta (2008), who found no significant effect of performance on satisfaction in his meta-analysis of 16 longitudinal studies from which 11 focused on task performance. This could be because none of the 11 meta-analyzed studies considered how the workers perceived their task performance: four studies measured task performance with objective indicators, while the other seven used subjective task performance evaluations by superiors. Incomplete feedback or self-evaluation bias could lead participants to perceive their task performance differently from their superiors or inconsistently with objective criteria. Good performance cannot enhance satisfaction if the worker is unaware they are performing well. The link between workers’ perception of their performance and their satisfaction (as measured in our experiment) should be different to the link between supervisors’ perception of workers’ performance and workers’ satisfaction (as measured in the meta-analysis). Our experiment uniquely manipulated perceived task performance and, unlike previous studies, captured the real impact of perceived performance on subsequent satisfaction.

Although we measured task satisfaction and task performance, our results can also help to understand the causality between overall job satisfaction and job performance as task satisfaction and task performance are very important dimensions of overall satisfaction and overall performance. Although our approach has limited environmental and construct validity and does not consider all dimensions of satisfaction and performance, the relationship between job satisfaction and job performance practically cannot be studied without shortcomings. We hope this experimental study with accurately measured important facets of satisfaction and performance may well complement other studies with higher construct environmental validity but lower measurement reliability, internal validity and with limited possibility to observe the causal effects.

### Strengths and Limitations

The laboratory conditions introduce some non-negligible limitations into our research. Participants did not complete the experimental tasks as part of their work responsibilities and were not rewarded for their performance. Therefore, the experimental context did not correspond to the context of usual work tasks, which reduces our results’ ecological validity. Participants’ careers were not dependent on their performance in the task; they participated voluntarily; and their motivation was not influenced by external stimuli (such as rewards or promotion). This created more space for the influence of inner motives than would likely be found within an organization. Consequently, the detected reciprocal impact of satisfaction on performance could be stronger in a laboratory experiment than under the conditions of a paid job. This might explain why we observed a somewhat stronger relationship between performance and satisfaction than those found in longitudinal (Riketta, 2008) and cross-sectional studies (Judge et al., 2001). Nevertheless, we think that the mechanism behind the satisfaction–performance relationship is the same under laboratory conditions and in an organization, although its effect might be suppressed or influenced by other variables in a non-laboratory context.

Moreover, laboratory conditions provided some advantages over cross-sectional and longitudinal studies. We were able to hold constant or even eliminate any possible intervening variables (e.g., organizational changes, relationship between employees, supervisor), which would be practically impossible in a real work environment. We were also able to measure participants’ performance through objective indicators providing greater accuracy than measurements in real organizations, with differences between task experiences, teams, regions, or customers. Even for employees in the same position, tasks are never exactly the same and performance ratings are always at least slightly biased.

Some notable limitations are also connected to the experimental design. A laboratory experiment can only focus on task performance in several tasks and does not consider contextual and long-term performance. Measuring task performance is much less complex than long-term measurement of job performance. Similarly, we only measured satisfaction with the task itself and neglected other components of job satisfaction, such as satisfaction with superiors. Therefore, our examination of the satisfaction–performance relationship focused only on the relationship between task performance and task satisfaction. Although these are important components of job performance and job satisfaction, the relationship between more complex job variables can differ. Therefore, our study’s results must be interpreted cautiously as evidence of causality among “task variables” that might explain causality among the “job variables” examined in dozens of cross-sectional (e. g. Heidemeier & Moser, 2019). and longitudinal studies (e. g., Eberegbe & Giovanis, 2020).

However, the experimental design is also a strength of our research. Riketta (2008) recommended experimental studies of the satisfaction–performance relationship, as such designs can provide clearer evidence on causality. By manipulating perceived performance and measuring satisfaction, we were able to clearly identify that people with higher perceived performance had higher task satisfaction than people with low perceived performance. As satisfaction with the new and unknown task was measured before participants first performed it, our study provides stronger support for the influence of performance on satisfaction than do previous longitudinal studies, where satisfaction in the first wave of measurement could have been affected by participants’ consistently good or bad performance.

Another limitation of our research is associated with the sample composition. Participants were mainly young, and the majority were university students. Since Kollmann et al. (2020) found that satisfaction-rewards relationship differs according to age recently, it seems possible that satisfaction-performance relationship can also be affected by age or work experience. Therefore, we recommend conducting another experiment with a different sample to strengthen our findings’ external validity.

Testing our hypotheses within one complex model can be considered a strength of our study. The repeated measurement of satisfaction and performance could easily produce a false positive result if a large number of partial analyses were performed. In addition, the complex model made it possible to monitor the indirect effects through multiple mediation analyses and accurately describe the nature of the relation between the monitored variables (e.g., the influence of perceived performance on objective performance in the third task through perceived performance in the second task, and not through satisfaction).

### Conclusion and Implications

The aim of this study was to explore causality of the satisfaction-performance relationship and to develop current understanding through an experiment with repeated measures of satisfaction and performance. Overall, it brings a new element to the mosaic of research into the satisfaction–performance relationship by reporting support for reciprocal causality between them. Specifically, the influence of perceived performance on subsequent satisfaction was more strongly supported than the influence of satisfaction on objective performance.

Since both worker satisfaction and performance are important for organizations, we recommended that they pay attention to worker satisfaction to boost their performance, making workers aware of their high performance in order to raise satisfaction. We encourage organizations to increase employee satisfaction through means besides positive performance feedback, as increasing satisfaction does not necessarily lead to performance growth. Employees satisfied with their job because they are performing well might become satisfied with their current efforts and approach, and so not pursue further improvement. We also recommend that researchers conduct longitudinal studies to monitor employees’ perceived task performance. It is unsurprising that the influence of performance on satisfaction has not been proven if the performance measurement differs from the worker’s own perception of their performance. For deeper understanding of the relationship, we recommend more experimental studies involving varying samples, different tasks, and the manipulation of various antecedents of satisfaction.
